# P-386. A Phase 4 Study to Evaluate the Safety and Efficacy of Oral B/F/TAF After Discontinuing Injectable CAB + RPV

**DOI:** 10.1093/ofid/ofaf695.603

**Published:** 2026-01-11

**Authors:** Sharon Walmsley, Moti Ramgopal, Thomas C S Martin, Jason Hindman, Hui Liu, Keith Aizen, Jason F Okulicz, Samir K Gupta

**Affiliations:** University Health Network, Toronto, Ontario, Canada; Midway Immunology and Research Center, Fort Pierce, Florida; University of California San Diego, La Jolla, California; Gilead Sciences, Foster City, California; Gilead Sciences, Inc., Foster City, CA, USA, Foster City, California; Gilead Sciences, Inc., Foster City, California; Gilead Sciences, Inc., Foster City, California; Indiana University School of Medicine, Indianapolis, IN

## Abstract

**Background:**

People with HIV-1 (PWH) on injectable cabotegravir + rilpivirine (CAB+RPV) may not stay on injectable antiretroviral therapy (ART) for various reasons. Given the long half-life and pharmacokinetic decay of CAB and RPV, switching to oral ART involves the overlap of ART agents. Bictegravir (BIC)/emtricitabine/tenofovir alafenamide (B/F/TAF) is a guideline-preferred once-daily oral regimen, but the overlap of the two integrase inhibitors, CAB and BIC, has not been evaluated.

**Figure:**
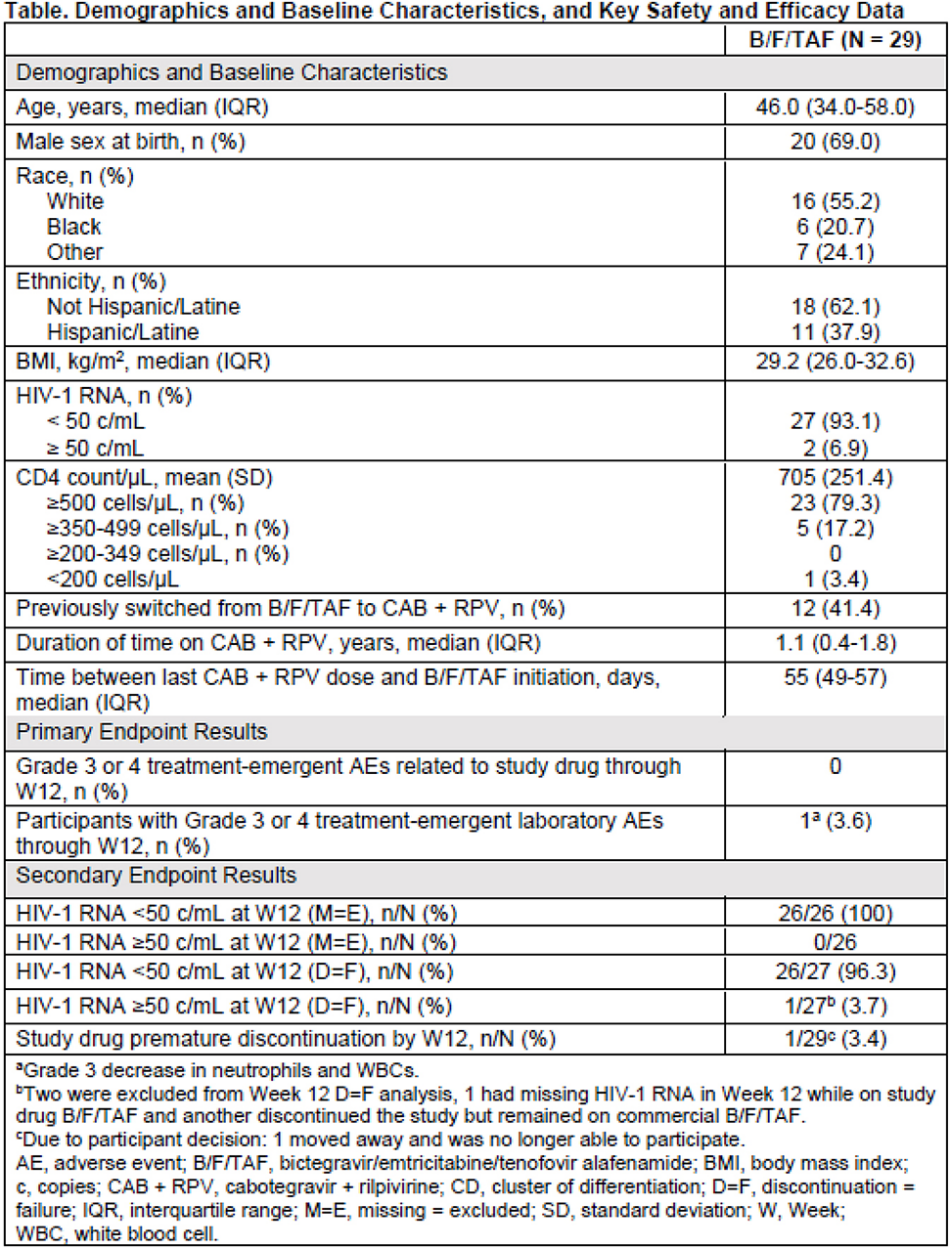

**Methods:**

This prospective, single-arm, open-label, interventional Phase 4 study (NCT06104306) included PWH who chose to switch from every-2-month CAB+RPV, having maintained HIV-1 RNA < 50 copies(c)/mL for ≥ 6 months at screening, to daily B/F/TAF due to intolerance, adverse events (AEs), or personal preference. Coprimary endpoints were the proportion of participants experiencing treatment-emergent Grade 3/4 study drug–related AEs and Grade 3/4 laboratory abnormalities through Week (W)12. Secondary endpoints included the proportion of participants with HIV-1 RNA ≥ 50 c/mL at W12 by missing = excluded (M=E) and discontinuation = failure (D=F) analyses, and the proportion of participants discontinuing B/F/TAF by W12.

**Results:**

Twenty-nine participants from North America switched to B/F/TAF around the time of their next scheduled CAB+RPV dose (Table). Median (IQR) age was 46 (34-58) years, 69% were assigned male at birth, 55.2% were White, 20.7% Black, and 37.9% Hispanic/Latine. The most common reason reported for switching from CAB+RPV to B/F/TAF was side effects (55.2%). No participants experienced treatment-emergent Grade 3/4 study drug–related AEs and 1 (3.6%) experienced treatment-emergent Grade 3/4 laboratory abnormalities (Grade 3 reduced neutrophil and total white blood cell counts), deemed unrelated to study drug, through W12. No participants had HIV-1 RNA ≥ 50 c/mL at W12 (M=E). One out of 27 participants (3.7%) had HIV-1 RNA ≥ 50 c/mL at W12 (D=F). One (3.4%) participant discontinued B/F/TAF by W12 for reasons other than efficacy/safety (they moved away).

**Conclusion:**

Switching from CAB+RPV to B/F/TAF was safe and efficacious in this study. The data support switching from injectable CAB+RPV to oral B/F/TAF when needed or desired.

**Disclosures:**

Sharon Walmsley, MSc, MD, FRCPC, Gilead Sciences, Inc.,: Advisor/Consultant|Gilead Sciences, Inc.,: Grant/Research Support|Gilead Sciences, Inc.,: Honoraria|Gilead Sciences, Inc.,: CME Speaking events|GSK: Grant/Research Support|Janssen: Grant/Research Support|Merck: Advisor/Consultant|Merck: Grant/Research Support|Merck: Honoraria|Merck: CME Speaking events|ViiV Healthcare: Advisor/Consultant|ViiV Healthcare: Grant/Research Support|ViiV Healthcare: Honoraria|ViiV Healthcare: CME Speaking events Moti Ramgopal, MD, AbbVie: Honoraria|Gilead Sciences, Inc.: Advisor/Consultant|Gilead Sciences, Inc.: Honoraria|Shionogi: Advisor/Consultant|ViiV: Advisor/Consultant|ViiV: Honoraria Thomas CS Martin, MD, Gilead Sciences, Inc.: Grant/Research Support Jason Hindman, PharmD, MBA, Gilead Sciences, Inc.: Employee|Gilead Sciences, Inc.: Stocks/Bonds (Private Company) Hui Liu, PhD, Gilead Sciences, Inc.: Employee|Gilead Sciences, Inc.: Stocks/Bonds (Private Company) Keith Aizen, MD, Gilead Sciences, Inc.: Employment|Gilead Sciences, Inc.: Stocks/Bonds (Private Company) Jason F. Okulicz, MD, Gilead Sciences, Inc.: Employee|Gilead Sciences, Inc.: Stocks/Bonds (Public Company) Samir K. Gupta, MD, Gilead Sciences, Inc.: Advisor/Consultant|Gilead Sciences, Inc.: Honoraria|ViiV Healthcare: Advisor/Consultant|ViiV Healthcare: Grant/Research Support|ViiV Healthcare: Honoraria

